# Case Report: Uremia secondary to acute pyelonephritis in a patient with type 2 diabetes mellitus

**DOI:** 10.3389/fmed.2026.1764853

**Published:** 2026-07-01

**Authors:** Zhaohui Xu, Fang Yin, Lixia Zhao, Qian Hou, Guiyan Han, Jianying Wang, Kunying Zhang

**Affiliations:** 1 School of Clinical Medicine, Shandong Second Medical University, Weifang, China; 2 Department of Nephrology, Weifang People’s Hospital, Shandong Second Medical University, Weifang, China; 3 Department of Pathology, Weifang People’s Hospital, Shandong Second Medical University, Weifang, China

**Keywords:** acute kidney injury, acute pyelonephritis, acute tubulointerstitial nephritis, anuria, case report, *Escherichia coli*, type 2 diabetes mellitus

## Abstract

We report a 58-year-old male patient with acute kidney injury (AKI), who eventually developed anuria secondary to acute tubulointerstitial nephritis (ATIN) following acute pyelonephritis. Our patient had a 10-year history of type 2 diabetes mellitus, with an admission glycated hemoglobin (HbA1c) level of 6.3% and no prior history of renal disease. Before admission, he presented with painless gross hematuria and fever. *Escherichia coli* was detected in the urine culture at the local hospital, and anti-infective normalized his temperature, but urine output progressively decreased to anuria. After transfer to our hospital, renal biopsy confirmed ATIN. Following treatment with glucocorticoids, the patient’s urine output gradually increased, and renal function gradually recovered. During subsequent follow-up, the patient achieved complete remission with a favorable prognosis. For patients with a history of type 2 diabetes mellitus who develop anuria without typical manifestations after acute pyelonephritis, early completion of relevant examinations, clear identification of the etiology, timely treatment and regarding medical follow-up are crucial to maximize the improvement of the patient’s prognosis.

## Introduction

*Escherichia coli* is one of the primary pathogenic bacteria responsible for acute pyelonephritis (1). Severe AKI induced by such infection is more common in elderly individuals and patients with chronic kidney disease (2). However, the development of anuric AKI is rare and usually associated with severe complications such as septic shock (1, 3). ATIN is an immune-mediated disease that primarily affects the renal tubules and interstitium, and constitutes one of the important causes of AKI (4, 5). Clinically, infection-induced ATIN lacks specific manifestations and can present as either oliguric or non-oliguric AKI, with anti-infective therapy generally serving as the mainstay of treatment (6). Patients with type 2 diabetes mellitus may develop diabetic neurogenic bladder (DNB) (7, 8), resulting in bladder dysfunction. Consequently, some of these patients may present with atypical bladder irritative symptoms when suffering from a urinary tract infection (9). Diabetic patients carry a higher risk of urinary tract infection and are more susceptible to severe renal complications such as pyonephrosis (10). Delayed treatment may increase the risk of surgical intervention (11), and a small proportion of patients with poor glycemic control may even experience iliac aneurysm due to aggravated infection (12). This case report describes a patient with history of type 2 diabetes mellitus who developed anuria secondary to ATIN following acute pyelonephritis, without septic shock. The patient attained complete recovery after definite diagnosis via renal biopsy and timely glucocorticoid intervention. This case may offer clinical implications for the diagnosis and management of similar cases.

## Case presentation

A 58-year-old male presented to our hospital with complaints of “gross hematuria for 3 days, fever, decreased urine output, and elevated serum creatinine for 2 days.” He had a 10-year history of type 2 diabetes mellitus, was taking metformin 0.5 g twice daily, and did not monitor his blood glucose regularly. He had no previous history of kidney disease. Three days prior to admission, the patient developed painless gross hematuria without obvious predisposing factors, with no significant urinary urgency, frequency, or dysuria. Two days before admission, he developed fever with a maximum temperature of 39.3 °C, accompanied by decreased urine output, fatigue, nausea, and watery stools. He was admitted to the local hospital, where relevant examinations revealed urine leukocyte (LEU) 3+, urine protein (PRO) +, urine occult blood (BLD) 3+, blood urea nitrogen (BUN) 20.58 mmol/L, and serum creatinine (Scr) 313.7 μmol/L. Blood routine examination showed a white blood cell (WBC) count of 14.02 × 10^9^/L, a neutrophil percentage (N%) of 91%, a red blood cell (RBC) count of 3.85 × 10^1^2^/L, hemoglobin (Hb) of 125 g/L, a platelet (PLT) count of 219 × 10^9^/L, and procalcitonin (PCT) of 181 ng/ml. *Escherichia coli* was detected in the urine culture. He received empirical anti-infective treatment with piperacillin/tazobactam 4.5 g every 12 h, and his body temperature returned to normal, but his urine output progressively decreased to anuria. On admission to our hospital, physical examination showed a body temperature of 36.0 °C, a pulse of 74 beats per minute, a respiratory rate of 18 breaths per minute, and a blood pressure of 112/74 mmHg. There was obvious percussion tenderness in the left renal area, and no other significant abnormalities were found.

Laboratory examinations on admission were as follows: WBC count 11.42 × 10^9^/L, N% 80.70%, RBC count 3.64 × 10^1^2^/L, Hb 115 g/L, PLT count 91 × 10^9^/L, PCT 100 ng/ml, interleukin-6 (IL-6) 51.25 pg/mL, and HbA1c 6.3%, Scr 606 μmol/L, and BUN 24.2 mmol/L. Peripheral blood smear showed occasional schistocytes. Coombs test results: direct antiglobulin test (DAT): polyspecific IgG+C3 (+), complement C3 (+), monospecific IgG (−). Other examinations, including thyroid function, anti-phospholipase A2 receptor antibody, anti-glomerular basement membrane antibody, anti-neutrophil cytoplasmic antibody, antinuclear antibody profile, anti-streptolysin O test, rheumatoid factor, immunoglobulin, immunofixation electrophoresis, complement C3+C4, galactomannan test, (1, 3)-β-D-glucan test, epidemic hemorrhagic fever antibody, stool culture and identification for *Salmonella* and *Shigella*, complement factor H, factor H antibody, a disintegrin and metalloproteinase with thrombospondin type 1 motifs 13 (ADAMTS13), and ADAMTS13 activity-inhibiting antibody, were all unremarkable.

Imaging examinations: Urinary system ultrasound revealed normal size of both kidneys, increased echogenicity of the bilateral renal parenchyma (suggestive of diffuse bilateral renal changes), and poor bladder filling. Color Doppler ultrasound of the left renal vein and fundus examination of both eyes showed no significant abnormalities.

**FIGURE 1:**
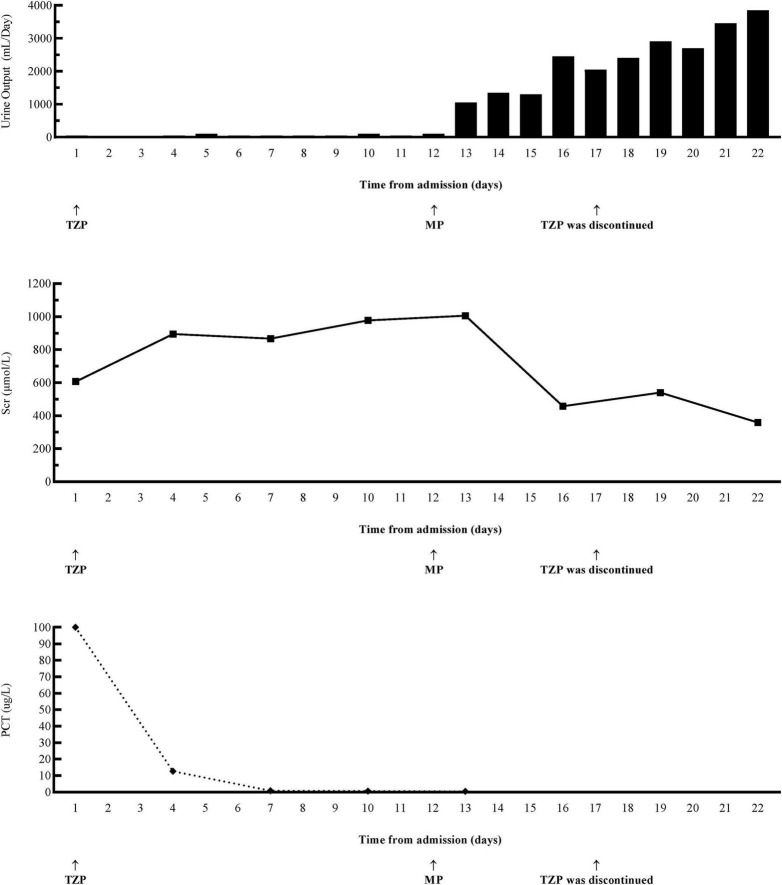
Changes in urine output and laboratory test results within 24 h during the patient’s hospitalization. Scr, Serum creatinine, PCT, procalcitonin, TZP, piperacillin/tazobactam 4.5 g, IV/12 h, MP, Methylprednisolone 40 mg, IV/day.

Admission diagnoses: Acute pyelonephritis, AKI, type 2 diabetes mellitus. The patient received empirical anti-infective treatment with piperacillin/tazobactam 4.5 g every 12 h, hemodialysis, and hypoglycemic therapy. The infection was gradually controlled, but the patient remained anuric (Figure 1).

**FIGURE 2:**
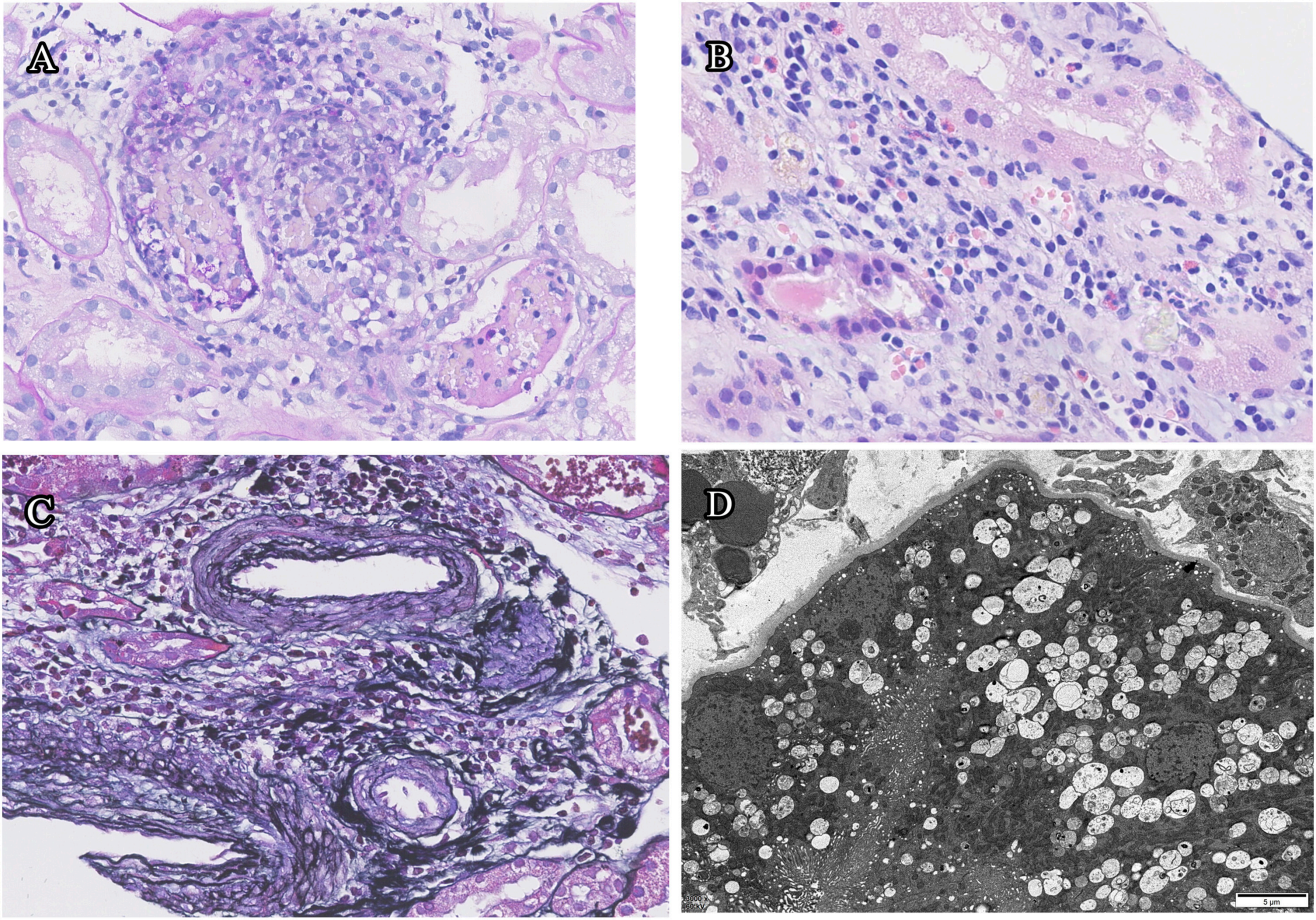
Pathological findings of the patient’s renal tissue. **(A)** Vacuolar and granular degeneration of renal tubular epithelial cells, with inflammatory cell casts observed in the tubular lumina (PAS staining, ×40). **(B)** Renal interstitial edema, showing infiltration of lymphocytes, monocytes, scattered neutrophils, a few plasma cells, and eosinophils (HE staining, ×40). **(C)** Thickening of small arterial walls, intimal fibroproliferative sclerosis, and no definite vascular endothelial injury (PASM staining, ×40). **(D)** Electron micrograph showing significant vacuolar degeneration of renal tubular epithelial cells with increased lysosomes; renal interstitial edema, accompanied by infiltration of a small number of lymphocytes, monocytes, and eosinophils as well as collagen fiber proliferation (electron microscopy, ×3000).

To clarify the cause of anuria, a percutaneous renal biopsy was performed on the 10th day after admission (Figure 2). Pathological findings of the renal biopsy tissue were as follows: A total of 19 glomeruli were observed. Mild segmental proliferation of glomerular mesangial cells and matrix was noted, along with vacuolar degeneration of the basement membrane. Renal tubular epithelial cells showed vacuolar and granular degeneration, multifocal brush border loss, epithelial cell flattening, segmental denuded basement membrane formation, and focal atrophy (approximately 10%). A small amount of protein casts, red blood cell casts, epithelial cell casts, inflammatory cell casts, and scattered urinary salt crystal deposits were detected in the tubular lumina. The renal interstitium exhibited edema, diffuse infiltration of lymphocytes and monocytes, scattered neutrophils, a few plasma cells and eosinophils, accompanied by fibrosis. Small arterial walls showed mild thickening, segmental hyalinization, and intimal fibroproliferative sclerosis; no definite vascular endothelial injury was observed. Congo red staining was negative. Immunofluorescence results: IgG: (−), IgA: (−), IgM: (±), C3: (−), C1q: (−), FRA: (−), κ chain and λ chain: (±), IgG1: (−), IgG2: (±), IgG3: (−), IgG4: (−). Electron microscopy revealed significant vacuolar degeneration of renal tubular epithelial cells with increased lysosomes. Renal interstitial edema was present, along with infiltration of a small number of lymphocytes, monocytes, and eosinophils, accompanied by collagen fiber proliferation. Pathological diagnosis: ATIN.

Based on the renal biopsy pathological findings, the patient received intravenous methylprednisolone 40 mg once daily on the 12th day after admission. Subsequently, the urine output gradually increased, and renal function improved progressively (Figure 1). On the 22nd day after admission, the patient’s serum creatinine decreased to 358 μmol/L, and he was successfully withdrawn from hemodialysis. After discharge, glucocorticoid therapy was switched to oral prednisone 40 mg once daily with regular medical follow-up. To prevent rebound phenomenon, glucocorticoids were maintained for 6 months and gradually tapered and discontinued according to the patient’s clinical condition. A follow-up assessment at 6 months after discharge revealed Scr 93 μmol/L and BUN 6.6 mmol/L. The last medical follow-up was performed at 9 months after discharge, Laboratory reexaminations showed: WBC count 7.30 × 10^9^/L, N% 57.3%, Scr 78 μmol/L, BUN 6.7 mmol/L. Urine routine examination revealed negative results for urine LEU, urine PRO, and urine BLD. The patient achieved complete recovery.

## Discussion

The patient was a middle-aged male with a history of type 2 diabetes mellitus. His clinical manifestations mainly included painless gross hematuria, fever, progressive elevation of serum creatinine, and rapid reduction of urine output to anuria. *Escherichia coli* was detected in the urine culture, and the renal pathological diagnosis was ATIN. Therefore, the patient was considered to have developed anuria due to ATIN secondary to *Escherichia coli* infection. After glucocorticoid treatment, the patient’s condition improved rapidly, and he was gradually withdrawn from hemodialysis. He was followed up in the outpatient clinic after discharge and achieved complete recovery.

Approximately 25%–60% of AKI episodes stem from prerenal causes, whereas AKI manifesting with oliguria or even anuria is more commonly attributed to urinary tract obstruction (13). Acute pyelonephritis is a common urinary tract infection, with clinical manifestations mainly including frequent micturition, urinary urgency, dysuria, lumbago, and fever. However, the development of AKI is rare (1). Severe AKI related to acute pyelonephritis often manifests as sepsis-associated hypotension and acute tubular necrosis, with a particularly high incidence in elderly individuals and patients with pre-existing chronic kidney disease (2). Oliguria or anuria frequently occurs as the infection advances to severe septic shock (1, 3). Our patient presented with progressive anuria on admission and had no other specific clinical manifestations. The patient had no prior history of kidney disease and showed no signs of septic shock. Persistent anuria remained after 10 days of anti-infective treatment, and the patient was dependent on hemodialysis. Therefore, performing renal biopsy to clarify the etiology and guide subsequent treatment is of great importance.

Pathologically, ATIN is characterized by interstitial edema, inflammatory cell infiltration, and tubulitis (4, 5). ATIN has multiple etiologies, among which infections, drugs, and toxins are the primary pathogenic factors. In our patient, *Escherichia coli* was detected in urine culture, and PCT levels were significantly elevated, which could indirectly indicate the diagnosis of infection-induced ATIN. Reports of anuria secondary to infection-associated ATIN are scarce in the literature (14–16). Li et al. proposed that after uropathogenic *Escherichia coli* (UPEC) invade the kidney, the bacteria adhere to the epithelial lining of renal collecting ducts. Some of the bacteria become embedded within neutrophil casts, and this subset may initiate neutrophil degranulation, leading to epithelial damage and subsequent tubular necrosis (17). Wang et al. have proposed that α-hemolysin (HlyA), a key toxin produced by *Escherichia coli*, forms pores in cell membranes leading to cellular lysis (18). Particularly at high concentrations, it directly damages renal epithelial cells, initiating tubular necrosis and inflammatory reactions (18). Furthermore, granulocyte-macrophage colony-stimulating factor (GM-CSF) can recruit monocytes to infiltrate the renal interstitium and promote their differentiation into pro-inflammatory M1 macrophages (18). These M1 macrophages secrete cytokines such as IL-6, interleukin-1β (IL-1β) and tumor necrosis factor-α (TNF-α), amplifying the inflammatory response and resulting in renal interstitial edema, tubular injury, and progressive deterioration of renal function (18). Our patient had an acute onset. Combined with markedly elevated PCT and IL-6 on admission and renal pathological findings (Figures 2A, B), we speculate on the pathogenesis as follows: upon invading the kidney, *Escherichia coli* may attach to the epithelial lining of the renal collecting ducts; some bacteria could be embedded in neutrophil casts and trigger neutrophil degranulation (17). Meanwhile, *Escherichia coli* releases large amounts of HlyA within a short time after renal invasion (18). Collectively, these alterations may contribute to epithelial damage as well as tubular necrosis and inflammatory reactions, which could eventually give rise to renal interstitial edema, tubular injury and rapid decline of renal function (17, 18), potentially leading to the occurrence of anuric AKI.

Some scholars argue that for infection-induced ATIN, anti-infective treatment is usually the mainstay, and glucocorticoid therapy has limited or no definite effect (6). Kwon et al. reported a case of megalocytic interstitial nephritis in a patient with acute pyelonephritis and oliguric acute kidney injury. Oliguria and renal dysfunction failed to improve after antibiotic therapy. Renal biopsy confirmed the diagnosis of megalocytic interstitial nephritis. Subsequent high-dose glucocorticoid treatment resulted in increased urine output and significant recovery of renal function, indicating that glucocorticoids are effective for infection-related interstitial nephritis when anti-infective therapy alone does not reverse oliguria (19). In our patient, anti-infective treatment and symptomatic supportive care were administered for more than 10 days after the onset of the disease; however, the patient remained persistently anuric and required hemodialysis. Following the confirmation of the renal pathological type and the initiation of glucocorticoid therapy, the patient’s condition improved rapidly and hemodialysis was successfully withdrawn. Praga and González have suggested that the prognosis of most patients with ATIN is poor, which may be associated with interstitial fibrosis caused by the rapid infiltration of inflammatory cells in the interstitium. Early administration of glucocorticoid therapy is conducive to the recovery of renal function (4). Combined with the subsequent treatment and reexamination results of our patient, early administration of glucocorticoid therapy can actively improve the prognosis and even achieve complete recovery.

Individuals with type 2 diabetes mellitus face an elevated risk of infections, and the urinary tract represents the most commonly affected site (10). The clinical spectrum of urinary tract infections (UTIs) in this population spans asymptomatic bacteriuria (ASB), lower urinary tract infections (cystitis), pyelonephritis, and severe urosepsis (10). Severe complications of UTIs—including emphysematous cystitis, emphysematous pyelonephritis, renal abscesses, and renal papillary necrosis—occur significantly more frequently in diabetic patients than in the general population (10). Chakit et al. reported a diabetic patient with recurrent urinary tract infections and multiple urolithiasis. Imaging workup demonstrated severe left hydroureteronephrosis, multiple calculi and a non-functioning left kidney. The patient ultimately underwent extraperitoneal nephroureterectomy as definitive treatment (11). Chakit et al. proposed that the concurrent presence of urolithiasis and diabetes can aggravate pyelonephritis and trigger the formation of massive pyonephrosis. In such scenarios where renal function is completely lost, nephrectomy constitutes the sole available treatment (11). A study on acute pyelonephritis conducted by Kim et al. demonstrated that compared with non-diabetic patients, diabetic patients had a significantly lower incidence of urinary urgency, frequency, and dysuria, along with a higher incidence of azotemia and a longer hospital stay (9). Poor glycemic control is a significant risk factor for the development of urosepsis in diabetic patients (20). Our patient regularly took metformin 0.5 g twice daily, and the admission HbA1c was 6.3%. The patient did not present with bladder irritative symptoms such as urinary urgency, frequency, or dysuria during the disease course. The possible underlying causes are speculated as follows: Firstly, it might be attributed to reduced urine output and poor bladder filling. Secondly, it may be associated with DNB. DNB is a common complication in diabetic patients and a manifestation of diabetic urogenital autonomic neuropathy (7, 8). Traditional viewpoints suggest that the typical symptoms of diabetic cystopathy include decreased bladder sensation, increased bladder capacity, and impaired bladder emptying (7, 8). Under normal circumstances, during the development of acute pyelonephritis, urine containing pathogenic bacteria enters the bladder through the ureters, triggering an inflammatory response that irritates the mucosa of the bladder neck and trigone, thereby inducing bladder irritative symptoms such as urinary urgency, frequency, and dysuria. Nevertheless, patients with diabetes mellitus tend to have declined bladder sensory function, which may result in weakened sensitivity to inflammatory stimuli in the setting of acute pyelonephritis. Such changes are likely to obscure typical urinary irritative manifestations, and the patients may merely manifest fever or lumbago.

In conclusion, some patients with type 2 diabetes mellitus may present with atypical bladder irritative symptoms and occult clinical manifestations when developing urinary tract infections, which can easily lead to misdiagnosis or missed diagnosis. If progressive oliguria or even anuria occurs, early percutaneous renal biopsy is recommended to clarify the etiology. Early diagnosis, timely treatment and regarding medical follow-up are crucial for the recovery of renal function in such patients.

## Data Availability

The original contributions presented in this study are included in this article/supplementary material, further inquiries can be directed to the corresponding author.
